# A 3-D finite element analysis of the effect of dental implant thread angle on stress distribution in the surrounding bone

**DOI:** 10.34172/joddd.2022.009

**Published:** 2022-05-29

**Authors:** Katayoun Sadr, Seyyed Mahdi Vahid Pakdel

**Affiliations:** Department of Prosthodontics, Faculty of Dentistry, Tabriz University of Medical Sciences, Tabriz, Iran

**Keywords:** Dental implants, Finite element analysis, Implant thread

## Abstract

**Background.** This study aimed to evaluate the effect of an increase in fixture thread face angle on the amount and distribution of stresses in the surrounding bone of implants with four different thread shapes by three-dimensional finite element analysis.

**Methods.** Eight different fixture designs, with v-shaped, buttress, reverse buttress, and trapezoid threads, and two face angles of 20 and 35 degrees, were modeled using a software program. Each model was affected by two static forces with different values and angles (200-N axial 0° force and 100-N 45° oblique force) to compare the distribution of stress in different fixture designs.

**Results.** The maximum von Mises stress was detected in v-shaped threads. An increase in the angle of the threads to 35° significantly decreased maximum von Mises stress in cortical bone in v-shaped and reverse buttress threads; however, the von Mises stress in the trapezoid and buttress threads increased with an increase in the thread angle.

**Conclusion.** Under the limitations of this study, although the shape of the thread and thread surface angle does not have a definite role in stress distribution in the bone surrounding the implant, they are effective in the amount and type of stress induced in the bone supporting the implant.

## Introduction

 The long-term and predictable success of dental implants has made them one of the main therapeutic options for replacing missing teeth.^1,2^ Dental implants support prostheses to replace missing teeth after osseointegration with jawbones, which means creating a functional and structural contact between viable osseous tissues and the implant surface without an intermediary connective tissue.^3^ As a result, the long-term success of implant therapy depends on establishing and maintaining proper implant stability in the jawbone.^4^ Various factors affect establishing successful osseointegration, including implant material, implant design, surface quality, bone status, surgical technique, and loading condition.^5^

 Of all the factors above, the design of the implant body, which is in direct contact with bone, is of great importance. Incorporating threads into the implant body increases its contact surface with bone,^6^ improving its mechanical contact with the bone and initial stability. In addition, forces are transferred from the implant to the surrounding bone through the implant and threads. Consequently, implant design and shape can play an important role in the secondary stability and biological contact between bone and implant and the success of osseointegration.^1,2^

 Therefore, numerous studies have investigated the geometry of threads, including thread depth, thread thickness, face angle, pitch, and helix angle, to help clinicians select the best shape of the implant for establishing and maintaining the long-term success of treatment.^7^

 Implants on the market have threads with different shapes, including v-shaped, square-shaped, buttress, reverse buttress, and spiral shape, with different face angles and thread widths.^6^ Thread face angle is the angle between the upper and lower surfaces of the thread and the line perpendicular to the long axis of the implant.^5^

 Occlusal forces on the prosthesis are transmitted through the implant body to the surrounding bone. Three types of forces might be created at the implant‒bone interface. Studies have shown that compressive forces have the most favorable effects on bone and increase its strength. In contrast, shearing forces have the most adverse effects on bone and compromise it.^8^ The shape of the implant and the thread angles can affect the direction of forces at the bone‒implant interface. In square and buttress threads, axial forces are mostly transmitted to the bone in the form of compressive forces,^9^ while axial forces in v-shaped and reverse-buttress threads are transmitted in a combination of shearing, compressive and tensile forces.^8^ Furthermore, shearing forces in different threads increase with an increase in thread face angle.

 In this study, three-dimensional finite element analysis was used to evaluate the effect of an increase in face angle on the amount and distribution of stresses in the surrounding bone of implants with v-shaped, trapezoidal, buttress, and reverse buttress threads with two angles of 20 and 35 degrees under axial and oblique forces, with similar thread geometry.

## Methods

**Figure 1:**
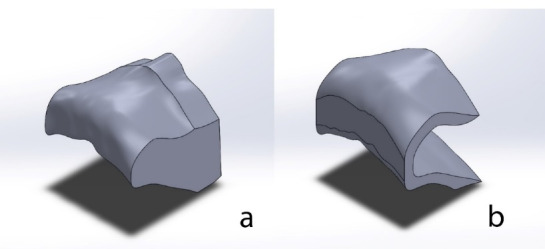
The bone block studied in the upper central incisor region (a). Cortical bone, measuring 1 mm in thickness (b).

 First, using cone-beam computed tomography images from a medium-sized dry skull and also by using Image Control System (CT-Scan Mimics: Materialise Interactive Medical Image Control System; Leuven, Belgium), a three-dimensional model of the maxillary bone was prepared; then a bone block from the anterior region of the maxilla was selected to study the upper central incisor region (Figure 1a).

 The alveolar process was cancellous bone, and its buccal and lingual aspects and crest were covered with cortical bone, measuring 1 mm in thickness (Figure 1b).

**Figure 2:**
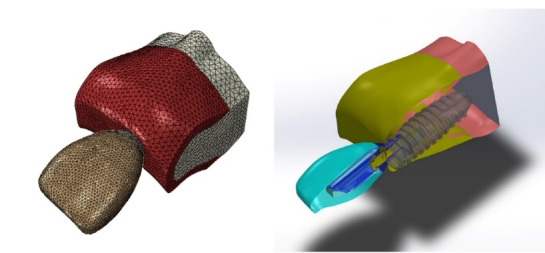
Implant model with the abutment, abutment screw, and crown in the anterior region of the maxilla.

 For modeling the implants and abutments, Nobel Biocare titanium cylindrical implants (Nobel Biocare Inc., Zürich-Flughafen, Switzerland) were used due to their extensive use in the clinic, with 12-mm length and 4-mm diameter and v-shaped threads. For abutment modeling, a straight Nobel Biocare abutment (Nobel Biocare Inc. Zürich-Flughafen, Switzerland) was used with a diameter of 4 mm, a height of 5 mm, a gingival height of 2 mm, and a 5º occlusal divergence. The abutment was placed on the fixture, and a metal‒ceramic crown of the upper central incisor was constructed with a mesiodistal width of 9 mm, a cervicoincisal height of 11 mm, and a labiopalatal thickness of 7 mm. The fixture, abutment, and crown were scanned with a 3-D scanner (ATOSII GOM, Braunschweig, Germany). The data obtained for software modeling were transferred to the Auto CAD software program (2010, Solid Works 2015, Rapid Form, 2006). Then the implant with abutment and abutment screw and crown were placed in a virtual state, directly and perpendicular to the horizontal plane within the bone (Figure 2).

**Figure 3:**
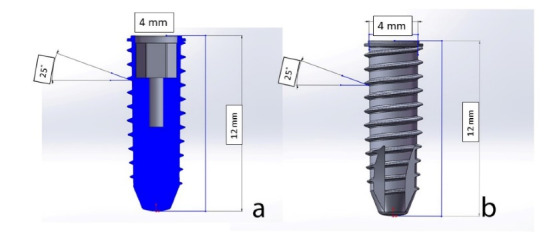
Schematic representation of the fixtures designed by the software program.

 Eight different designs of fixtures, with v-shaped, buttress, reverse buttress, and trapezoid threads with two face angles of 20 and 35 degrees, were modeled using the software program. Figure 3 presents the specifications of the designed fixtures.

 The models prepared in the Solid Works modeling software program were transferred to ABAQUS Ver 6.9 analytical software program (Abaqus FEA, ABAQUS Inc.) after assembling and finalizing the model. Then the meshing procedure of the models was carried out with the software program using eight-point or ten-point solid elements.

 The contact between the cancellous and cortical bones and the implant was considered complete osseointegration, and the contact between the implant and the abutment was also modeled as a rigid contact. All the final nodes of the alveolar bone in the model were considered fixed and free of motion in all directions and as borderline conditions.

**Figure 4:**
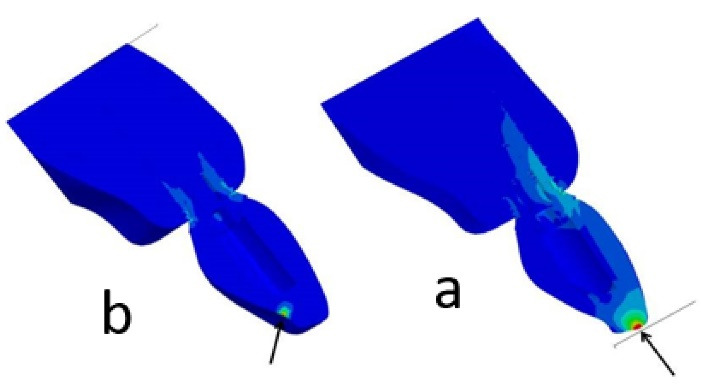
Force application location: Axial load (a), oblique load (45°) (b).

 The models were affected by two static forces with different values and angles to compare stress distribution in different fixture designs (Figure 4).

1. 200-N axial force with a 0° angle relative to the incisal edge of the crown
2. 100-N oblique force with a 45° angle relative to a point 2 mm below the incisal edge of the crown on the palatal surface

All the materials used in the above evaluation were considered homogenous and isotropic, and their mechanical properties were derived from valid references, presented in Table 1.^10^

**Table 1:** Mechanical properties of materials used in finite element model

**Materials**	**Young’s modulus (GPa)**	**Poisson ratio**
Cortical bone	14	0.3
Cancellous bone	1.37	0.3
Titanium	110	0.35
Nickel‒chromium alloy	218	0.33
Feldspathic porcelain	69	0.3

## Results

**Figure 5:**
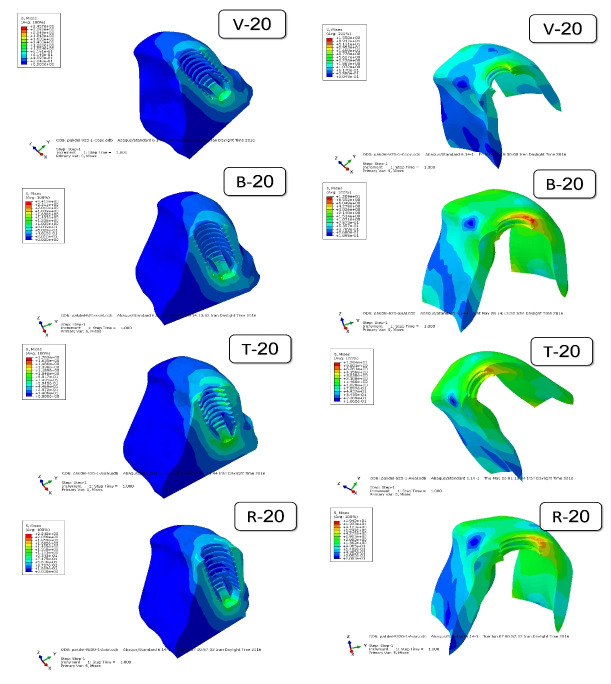
Distribution of stresses around the implant with a face angle of 20° under an axial force. Left: cancellous bone; right: cortical bone.

**Figure 6:**
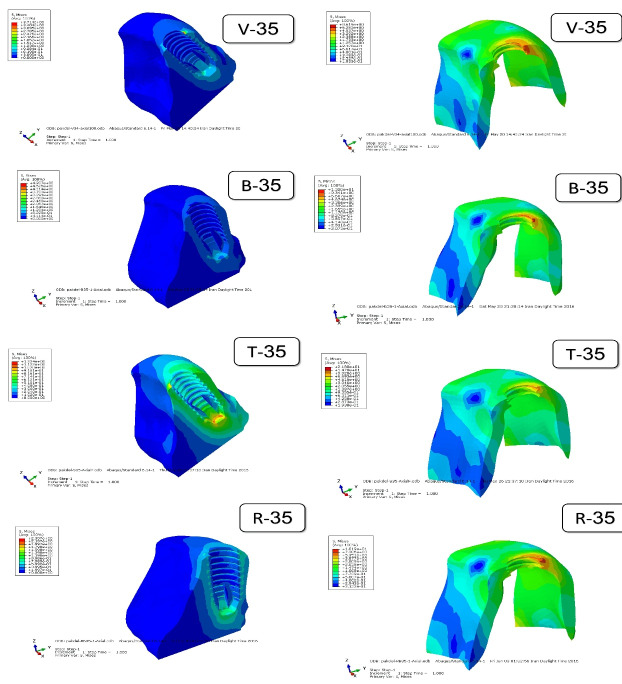
Distribution of stresses around the implant with a face angle of 35° under axial forces. Left: cancellous bone; right: cortical bone.

**Figure 7:**
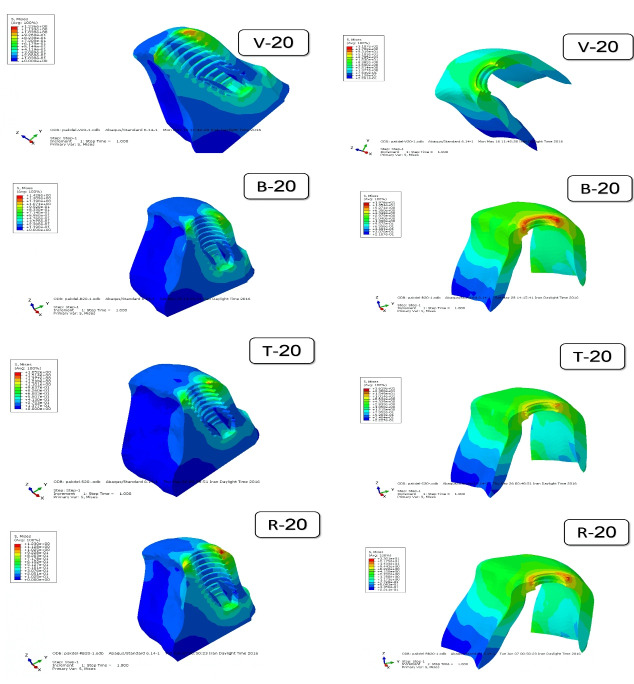
Distribution of stresses around the implant with a face angle of 20° under oblique forces with an angle of 45°. Left: cancellous bone; right: cortical bone.

**Figure 8:**
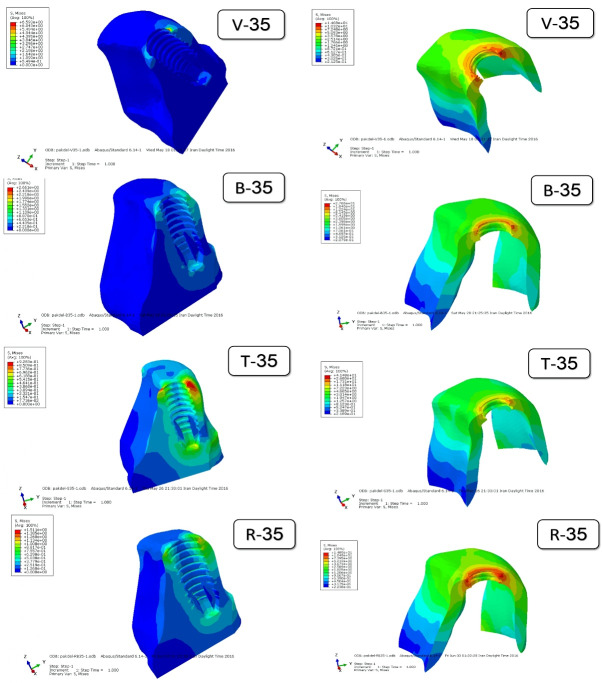
Distribution of stresses around the implant with a face angle of 35º under oblique forces with an angle of 45°. Left: cancellous bone; right: cortical bone.

Figures 5 to 8 present the distribution and maximum von Mises stresses (Gpa) by using color codes from blue to red, indicating minimum stress values to maximum stress values, respectively,^11,12^ in the mesiodistal cross-sections of the implants with different designs of threads and in the surrounding cortical and cancellous bone under axial and oblique forces.

Figure 5 shows the distribution of stress in the cortical and cancellous bone around the implant with v-shaped (V), buttress (B), trapezoid (T), and reverse buttress (R) threads with a face angle of 20º. Figure 6 shows the same specimens with a 35º face angle. Comparison of the figures shows that although the maximum von Mises stress in the specimens was different under different forces, the distribution of stress followed a similar pattern. Maximum von Mises stress was seen in the crest cortical bone along the first and second threads. The stress level in the cancellous bone was less than that in the cortical bone.

Figures 7 and 8 show the distribution of stresses in the cortical and cancellous bone around the samples under 45° force applied 2 mm below the incisal edge. Maximum von Mises stress was seen in the crest cortical bone along the first and second threads. The stress level in cancellous bone was less than that in cortical bone.

Tables 2 to 5 present maximum von Mises stresses and compressive, shearing, and tensile forces under axial and oblique forces in the samples.

**Table 2:** Maximum von Mises stress in the cancellous and cortical bone around implants with different designs of threads under axial and oblique forces

**Surface angle**	**Axial load**		**Oblique load (45°)**	
	**Maximum von Mises (Gpa)** **cancellous bone**	**Maximum von Mises (Gpa)** **cortical bone**	**Maximum von Mises (Gpa)** **cancellous bone**	**Maximum von Mises (Gpa)** **cortical bone**
V-shaped (20°)	2.45	155.0	1.23	310.7
V-shaped (35°)	3.71	8.61	6.59	14.68
Buttress (20°)	2.41	12.09	1.42	19.76
Buttress (35°)	4.93	13.20	2.66	27.66
Trapezoid (20°)	1.78	12.94	1.65	36.19
Trapezoid (35°)	1.22	21.80	0.92	41.48
Reverse (20°)	2.24	19.49	1.23	33.01
Reverse (35°)	2.39	10.19	1.51	14.89

**Table 3:** Maximum compressive stresses in the cancellous and cortical bone around implants with different designs of threads under axial and oblique forces

**Surface angle**	**Axial load**		**Oblique load (45˚)**	
	**Compressive stress (Gpa)** **cancellous bone**	**Compressive stress (Gpa)** **cortical bone**	**Compressive stress (Gpa)** **cancellous bone**	**Compressive stress (Gpa)** **cortical bone**
V-shaped (20°)	1.87	226.8	1.01	447.5
V-shaped (35°)	2.17	10.34	4.46	17.60
Buttress (20°)	2.02	12.96	2.21	24.28
Buttress (35°)	2.15	15.17	1.06	26.27
Trapezoid (20°)	2.37	12.84	1.73	35.74
Trapezoid (35°)	1.49	21.12	1.15	40.77
Reverse (20°)	2.03	17.68	1.26	29.21
Reverse (35°)	2.22	11.43	1.26	17.68

**Table 4:** Maximum shearing stresses in the cancellous and cortical bone around implants with different designs of threads under axial and oblique forces

**Surface angle**	**Axial load**		**Oblique load (45˚)**	
	**Shear stress (Gpa)** **cancellous bone**	**Shear stress (Gpa)** **cortical bone**	**Shear stress (Gpa)** **cancellous bone**	**Shear stress (Gpa)** **cortical bone**
V-shaped (20°)	0.29	3.30	0.34	6.44
V-shaped (35°)	0.22	1.68	0.30	4.84
Buttress (20°)	0.28	2.62	0.43	5.44
Buttress (35°)	0.39	3.06	1.15	5.56
Trapezoid (20°)	0.13	2.28	0.17	4.41
Trapezoid (35°)	0.19	1.95	0.18	5.51
Reverse (20°)	0.29	1.73	0.35	5.12
Reverse (35°)	0.27	1.86	0.39	5.68

**Table 5:** Maximum tensile stresses in the cancellous and cortical bone around implants with different designs of threads under axial and oblique forces

**Surface angle**	**Axial load**		**Oblique load (45˚)**	
	**Tensile stress (Gpa)** **cancellous bone**	**Tensile stress (Gpa)** **cortical bone**	**Tensile stress (Gpa)** **cancellous bone**	**Tensile stress (Gpa)** **cortical bone**
V-shaped (20°)	2.26	32.48	1.45	62.48
V-shaped (35°)	2.40	11.23	3.12	13.44
Buttress (20°)	2.90	8.48	1.59	21.07
Buttress (35°)	3.59	12.10	2.25	27.63
Trapezoid (20°)	2.10	7.20	1.24	13.27
Trapezoid (35°)	1.64	6.58	8.62	24.79
Reverse (20°)	2.03	8.32	1.47	27.43
Reverse (35°)	1.85	7.20	1.56	15.87

 The maximum von Mises stress was detected in v-shaped threads followed by reverse threads with a 20° angle, which was higher than that in trapezoid and buttress threads. An increase in the angle of the threads to 35° resulted in a significant decrease in maximum von Mises stress in cortical bone in v-shaped threads, which was the minimum stress in the cortical bone between different threads.

 von Mises stress in the cortical bone and in the reverse threads also decreased with an increase in the thread angle; however, the von Mises stress in trapezoid and buttress threads increased with an increase in the thread angle. Changes in von Mises stress followed the same pattern in threads with an angle of 20° under oblique forces.

Tables 3 to 5 show that compressive stresses applied to cortical bone were higher than shearing and tensile stresses. The samples with a 20° angle and v-shaped and reverse threads underwent the highest compressive stresses. An increase in the angle to 35º resulted in increased compressive stresses in trapezoid and buttress threads and a decrease in these stresses in v-shaped and reverse threads.

 The maximum shearing stresses in samples with a 20° angle were recorded in descending order in v-shaped, buttress, trapezoid, and reverse threads. An increase in the angle to 35° resulted in an increase in shearing stresses in the buttress and reverse threads and in a decrease in these stresses in the v-shaped and trapezoid threads.

 The maximum and minimum tensile stresses were observed in samples with a 20° angle in v-shaped and trapezoid threads, respectively. As the angle increased, the tensile stress increased in the buttress threads and decreased in the three other threads.

## Discussion

 The occlusal forces are transmitted by the dental implants to the biological tissues surrounding them. Therefore, the aim of designing implants is to control the transfer and distribution of biomechanical forces to improve the function of implant-supported prostheses.^8^

 The finite element analysis is an effective tool for simulating clinical conditions to solve complex problems^13^; it is one of the best methods for examining the biomechanical behavior of implants under different forces.^14^ With this technique, it is possible to evaluate tensile, compressive, and shearing stresses separately and evaluate a combination of them, referred to as equivalent von Mises stress.^15^ In this study, the above technique was used, by standardizing all the other geometric parameters, to determine the effect of the shape and angle of the threads on the distribution and transfer of forces to the adjacent supporting tissues. von Mises, compressive, and shearing stresses were used to present the results.

 Consistent with some previous studies, the present study showed that although the maximum von Mises stresses were different in threads with different shapes, the distribution of stress was the same in all of them and followed a similar pattern.^2,4,7,10,15-17^ The maximum von Mises stress was detected in the cortical crest bone along the first and second threads, with higher stress in the cortical bone than the cancellous bone.^18^

 In this study, a comparison of maximum von Mises, compressive, and shearing stresses in various samples confirmed the effect of shape and surface angles of the threads in the transfer and the type of stresses exerted on the bone. Changing the surface angle from 20° to 35° in v-shaped and reverse threads resulted in a decrease in maximum von Mises stress in cortical bone and increased these stresses in trapezoid and buttress threads.

 A study by Kong et al,^10^ too, indicated the role of various surface angles in the magnitude and distribution of von Mises stress in v-shaped, reverse, square, and buttress threads. The study above also showed that the most suitable angle of thread in terms of the effect on the amount of stress in bone is not the same in all types of threads, consistent with the present study.

 Abangah et al^16^ also showed the role of the thread angles in the transfer and accumulation of stress in bone. The study showed that by increasing the angle of triangular and trapezoid threads, the maximum von Mises stress decreased, consistent with the present study concerning triangular threads but different from those concerning trapezoid threads, which might be attributed to the angles evaluated in the present study.

 NarendraKumar et al^19^ compared v-shaped implants with thread angles of 20, 30, 45, and 60 degrees and reported that 20° threads transmitted the maximum von Mises stresses to the bone. An increase in the angle from 30 to 45 degrees resulted in a decrease in stresses. However, an increase in the angle to 60° increased the stresses, consistent with the present study that indicated a decrease in stress by changing the angle in v-shaped threads from 20 to 35 degrees.

 According to Mosavar et al,^4^ the type of stress transmitted to the bone depends on the shape of the thread, and a change in thread surface angles resulted in a change in the type of stress in the bone surrounding the implant. An implant with an ideal design should be able to modify tensile and compressive forces and minimize shearing forces.^4^ Tensile forces act as a bone maintenance stimulus; therefore, the high accumulation of shearing forces, in association with the absence of sufficient compressive forces to stimulate the bone mechanically, might be the main etiologic factor for bone loss at the bone–implant contact area.^8^

 In the present study, the compressive stress in the cortical bone along the first and second threads was higher than the shearing and tensile stresses in all the samples, which is favorable. Among the examined specimens, the reverse threads with angles of 20 and 35 degrees and the trapezoid threads with an angle of 35 degrees were the most favorable, and the v-shaped and buttress threads with an angle of 35 degrees exhibited the least favorable behavior under the applied forces.

 The results of the present study support the results reported by Eraslan and Inan,^15^ indicating that apart from the variability of compressive stresses in the bone around different implants, the greatest compressive stress was observed around reverse threads. Therefore, this thread design can be useful for establishing bone around the implant. Furthermore, the results of this study were also consistent with those reported by Mosavar et al,^4^ who showed that the amount of compressive stress around the implants with reverse thread was higher than other threads; therefore, it is the most favorable form.

 According to the results of previous studies and the present study, each thread form has its own optimal thread surface angle. Therefore, we cannot consider an angle the most suitable angle in terms of distribution and transmission of forces in all the thread forms. The v-shaped, buttress, trapezoid, and reverse buttress threads do not necessarily have the same behavior under the occlusal forces, and the best angle for each thread shape should be obtained by comparing different angles.

## Conclusion

 Under the limitations of this study, although the shape of the thread and thread surface angle did not have a role in stress distribution in the bone surrounding the implant, they affected the amount and type of stress induced in the bone supporting the implant.

## Acknowledgments

 This study was supported by the Research Council of Tabriz University of Medical Sciences. The authors would like to thank the Council for its assistance in carrying out this study.

## Authors’ Contributions

 KS collected initial data, reviewed the relevant articles, prepared the manuscript, and edited the manuscript. SMVP prepared the subject proposal, performed the study, and prepared the manuscript.

## Funding

 This study was funded by the Research Council of Tabriz University of Medical Sciences.

## Ethics Approval

 No need for ethics approval because of the in vitro nature of the study.

## Competing interests

 None declared.
